# L-valine derived from the gut microbiota protects sepsis-induced intestinal injury and negatively correlates with the severity of sepsis

**DOI:** 10.3389/fimmu.2024.1424332

**Published:** 2024-07-04

**Authors:** Yifan Chen, Keyuan Sun, Yue Qi, Jianguo Tang, Haiyan Zhu, Zetian Wang

**Affiliations:** ^1^ Department of Trauma-Emergency & Critical Care Medicine, Shanghai Fifth People’s Hospital, Fudan University, Shanghai, China; ^2^ Department of Biological Medicines, Shanghai Engineering Research Center of ImmunoTherapeutics, School of Pharmacy, Fudan University, Shanghai, China

**Keywords:** gut microbiota, intestinal injury, L-valine, microbiota metabolites, sepsis

## Abstract

**Background:**

The protective role of gut microbiota and its metabolites against intestinal damage in sepsis patients remain unclear.

**Methods:**

Fecal samples were acquired from patients categorized into sepsis and non-sepsis groups for analysis of microbial composition via 16S rRNA sequencing and untargeted metabolomics analysis. We assessed the impact of gut microbiota from sepsis patients on intestinal barriers in antibiotic-treated mice. Furthermore, We conducted spearman’s correlation analysis to examine the relationship between metabolites and the severity of sepsis. Additionally, we performed animal experiments to validate the functionality of identified metabolites.

**Results:**

The diversity of intestinal flora is decreased in patients with sepsis compared to the control group. Through fecal microbiota transplantation experiments, it was discovered that the gut microbiota derived from sepsis patients could induce intestinal damage in antibiotic-treated mice. Metabolomics analysis of the microbiota revealed a significant enrichment of the Valine, leucine, and isoleucine biosynthesis pathway. Further analysis showed a significant decrease in the abundance of L-valine in sepsis patients, which was negatively correlated with APACHE-II and SOFA scores. In sepsis mouse experiments, it was found that L-valine could alleviate sepsis-induced intestinal damage.

**Conclusion:**

Alterations in microbial and metabolic features in the gut can affect the severity of sepsis. Furthermore, L-valine can protect against sepsis-induced intestinal injury.

## 1 Introduction

Sepsis is a common lethal condition that affects patients in the intensive care unit, and is associated with a high risk of mortality (1, 2). The sepsis-induced inflammatory response leads to significant damage to the intestines and subsequent bacterial translocation, thereby causing the development of systemic infections and eventually resulting in death (3–5). However, the protective effects on the function of the intestinal barrier are uncertain, making it imperative to urgently discover a new approach that can effectively safeguard and preserve the integrity of the intestinal barrier in cases of sepsis (6).

Dysbiosis of gut microbiota directly disrupt the metabolism of microbiota, thus damaging the intestinal barrier. As a result, bacterial endotoxins are released, leading to the persistence and exacerbation of inflammation (1, 7). The microbiome, especially the gut microbiome, produces a myriad of active metabolites (8). For example, gut microbial metabolites like short-chain fatty acids, secondary bile acids, and trimethylamine-N-oxide interact with host receptors such as G protein-coupled receptors, affecting host metabolic processes. They can significantly affect physiological homoeostasis, which caused metabolic diseases (9, 10). However, the protective role of gut microbiota and its metabolites against intestinal damage in sepsis patients remain unclear and require further investigation.

In this study, 16S rRNA sequencing and metabolomic analyses were used to analysis the fecal samples collected from sepsis patients and no-sepsis controls. The protective function of gut microbiota metabolites against sepsis-induced intestinal injury using animal experiments. In addition, we investigated the potential protective of gut microbiota metabolites through animal experiments. These provides a new therapeutic target for the prevention of intestinal injury in sepsis.

## 2 Materials and methods

### 2.1 Clinical samples collection

Fecal samples were gathered from sepsis patients and no-sepsis controls (n=13 each) at Shanghai Fifth People’s Hospital. The research adhered to the ethical standards and directives stipulated by the Human Research Ethics Committee of the hospital, under the approval document numbered 2019–118. The patients or their legal guardians provided informed consent before sample collection. The collected samples were immediately transferred into polyethylene tubes and preserved at -80°C for further analysis. Inclusion criteria for the sepsis group were: (i) Sepsis patients diagnosed following the Third International Consensus Definitions for Sepsis and Septic Shock (11); (ii) Patients in the age range of 18 to 80 years; (iii) Patients who were admitted to the department within 12 h following the onset of sepsis. The exclusion criteria were as follows: Patients diagnosed with hematologic malignancies, those with HIV infection, or anyone who had received antibiotics or immunosuppressive therapy within the last month; additionally, pregnant or breastfeeding individuals were not included in the study.

Inclusion criteria for the control group included: (i) individuals whose age and gender profiles aligned with those of the sepsis patient cohort; (ii) subjects with normal biochemical indices as established by routine medical examinations. Exclusion criteria for individuals in the control group included those with a history of sepsis, serious infections, hematologic malignancies, solid tumors, or inflammatory diseases (12). Illness severity was assessed with the Acute Physiology and Chronic Health Evaluation-II (APACHE-II) score along with the Sepsis-related Organ Failure Assessment (SOFA) score.

### 2.2 16S rRNA sequencing

QIAamp® Fast DNA Stool Mini Kit was used to extract DNA from fecal samples. The samples underwent 16S rRNA sequencing via Genesky Biotechnologies Inc. (Shanghai, China). Notably, appropriate primers (V4) were used to amplify the bacterial 16S rDNA hypervariable region 4. Libraries were mixed and subjected to quality control (QC) analyses, followed by paired-end sequencing via an Illumina Miseq instrument. Sequencing data and operational taxonomic units (OTUs) were used for downstream analyses. Alpha diversity metrics were derived from a consistently depth-normalized OTU table to guarantee equitable and precise comparisons. Beta diversity was assessed using the Bray-Curtis algorithm and unweighted UniFrac distances, facilitating the comparison of potential gut microbiota variability amongst groups. Finally, Principal Component Analysis (PCA) was used to determine significant differences in gut microbiota composition. The LEfSe (Linear Discriminant Analysis Effect Size) algorithm was employed to evaluate differences in taxonomic abundance across groups. To ascertain significance, we applied a threshold of a False Discovery Rate (FDR) below 0.05 for the Kruskal-Wallis test, coupled with a minimum logarithmic LDA (Linear Discriminant Analysis) score of 2.0.

### 2.3 Metabolomics analyses

Fecal extracts were produced by adding cold water (500 mL) to 100 mg of fecal specimens, followed by vortexing and centrifugation at 13,000 rpm and 4°C for 15 min. The samples were then filtered with a 0.22 um filter and stored at -80°C for LC-MS analysis. The pooled samples were routinely used to create QC samples in equal amounts to ensure platform stability throughout the study. Metabolite profiles were analyzed through AB Sciex AB TripleTOF 6600 mass spectrometer with ESI sources in both positive and negative ion modes under TOF settings similar to those in previous studies. All analyses were conducted using HPLC-grade reagents. Progenesis QI software (Waters Corporation, MA, USA) was used to analyze LC-MS data. Metabolite identification was conducted utilizing Progenesis QI software for data processing. Group metabolic variations were pinpointed using ellipses defined by the Hotelling T2 area, with a 95% confidence threshold serving as the demarcation for significance.

### 2.4 Mouse model experiments

**Figure 1:**
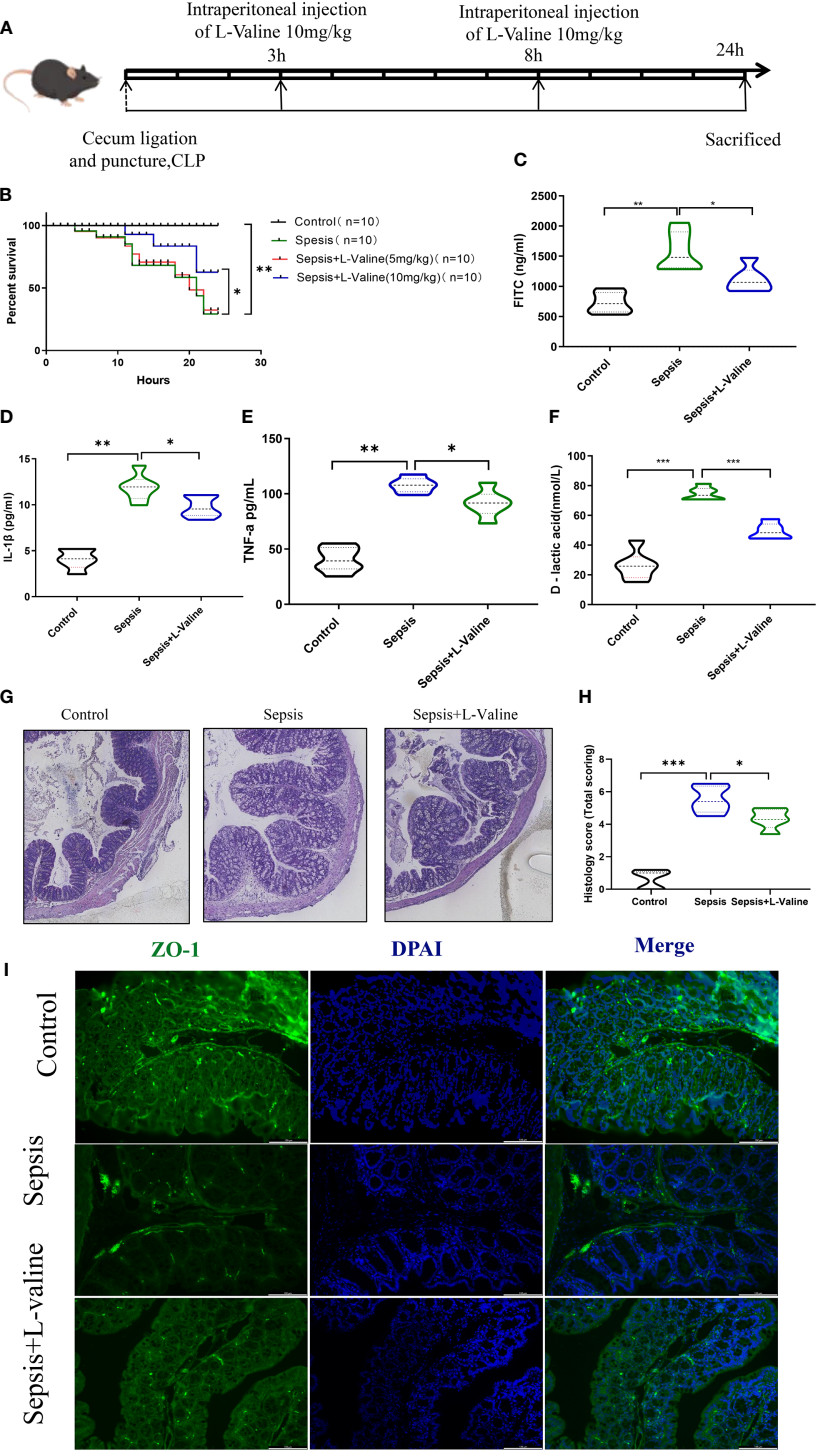
L-valine protects against sepsis-induced intestinal damage in mice. Experiments were conducted in mice to investigate the function of L-valine in protecting against sepsis-induced intestinal damage. **(A)** Design of animal experiment. **(B)** Kaplan-Meier curves demonstrating the survival of mice. **(C–F)** Levels of serum FITC **(C)**, IL-1β **(D)**, TNF-a **(E)** and D-lactic acid **(F)** levels were analyzed. **(G, H)** H&E staining. **(I)** ZO-1 was detected via immunofluorescent staining. *P < 0.05, **P < 0.01.

Male C57BL/6 mice, aged 6–8 weeks and weighing 20–23 g, obtained from the Animal Center of East China Normal University, were housed in conditions with regulated 12-hour light-dark cycles. The mice had free access to food and water. Animal experiments were approved by the Experimental Animal Ethical Review Committee of East China Normal University. In male C57BL/6 mice, sepsis was elicited via cecal ligation and puncture (CLP) surgical procedure. The mice were then randomly assigned into Control, Sepsis, Sepsis+L-valine groups (5 mg/kg and 10 mg/kg) (10 mice in each group). L-Valine (Abmole, M10525) was dissolved in a vehicle containing 10% DMSO and 90% saline [1:9], then intraperitoneally injected into the mice after 3 h and 8 h of the surgery. Subsequently, 24 hours after the surgery, fresh samples of stool, blood, and primary organs were gathered for additional examination, as illustrated in **Figure 1A**.

### 2.5 Fecal microbiota transplantation experiment

To investigate the impact of gut microbiota on differential susceptibility to sepsis, 6-to 8-week-old male C57BL/6J mice were purchased from the same batch of the same manufacturer and housed in conditions with regulated 12-hour light-dark cycles. and with a temperature range of 18–29°C, a daily temperature difference of ≤ 3°C, a relative humidity of 40%~70%, 10 fresh air exchanges per hour, an airflow velocity of ≤ 0.18m/s, a pressure difference of 25Pa, a cleanliness level of 10000, and an ammonia concentration of 15mg/m³, Noise ≤ 60dB, illuminance 150–300Lux. Antibiotics (ABX) (vancomycin, 100 mg/kg; neomycin sulfate 200 mg/kg; metronidazole 200 mg/kg; and ampicillin 200 mg/kg) were then given by gavage for 1 week to deplete the gut microbiota and create pseudosterile mice (13)=.

Collect 50 g of fresh faeces from the septic(n=13) and non-septic patients(n=13) described above, seal in sterile containers, place faeces samples on ice, and quickly transfer to a sterile biosafety cabinet. Add 250 ml sterile physiological saline water to the sample, stir well, and pass through stainless steel filters with pore sizes of 2.0 mm, 1.0 mm, 0.5 mm and 0.25 mm in sequence. Remove undigested food and small particulate matter from fecal fluid. After centrifugation at 6000 rpm for 15 minutes through a 0.25 mm pore size of fecal bacteria solution, the supernatant was discarded and the precipitate was resuspended in half the original volume of sterile physiological saline. Finally, the precipitate was diluted with 0.1M PBS (pH=7.2) containing 10% sterile medical glycerol, dispensed into cryotubes and transferred to a refrigerator at -80°C for 1–8 weeks, within 2 hours of collection of the stool sample. Before fecal bacteria transplantation, the fecal bacteria solution was taken out and then cooled for 2 hours to dissolve.

**Figure 2:**
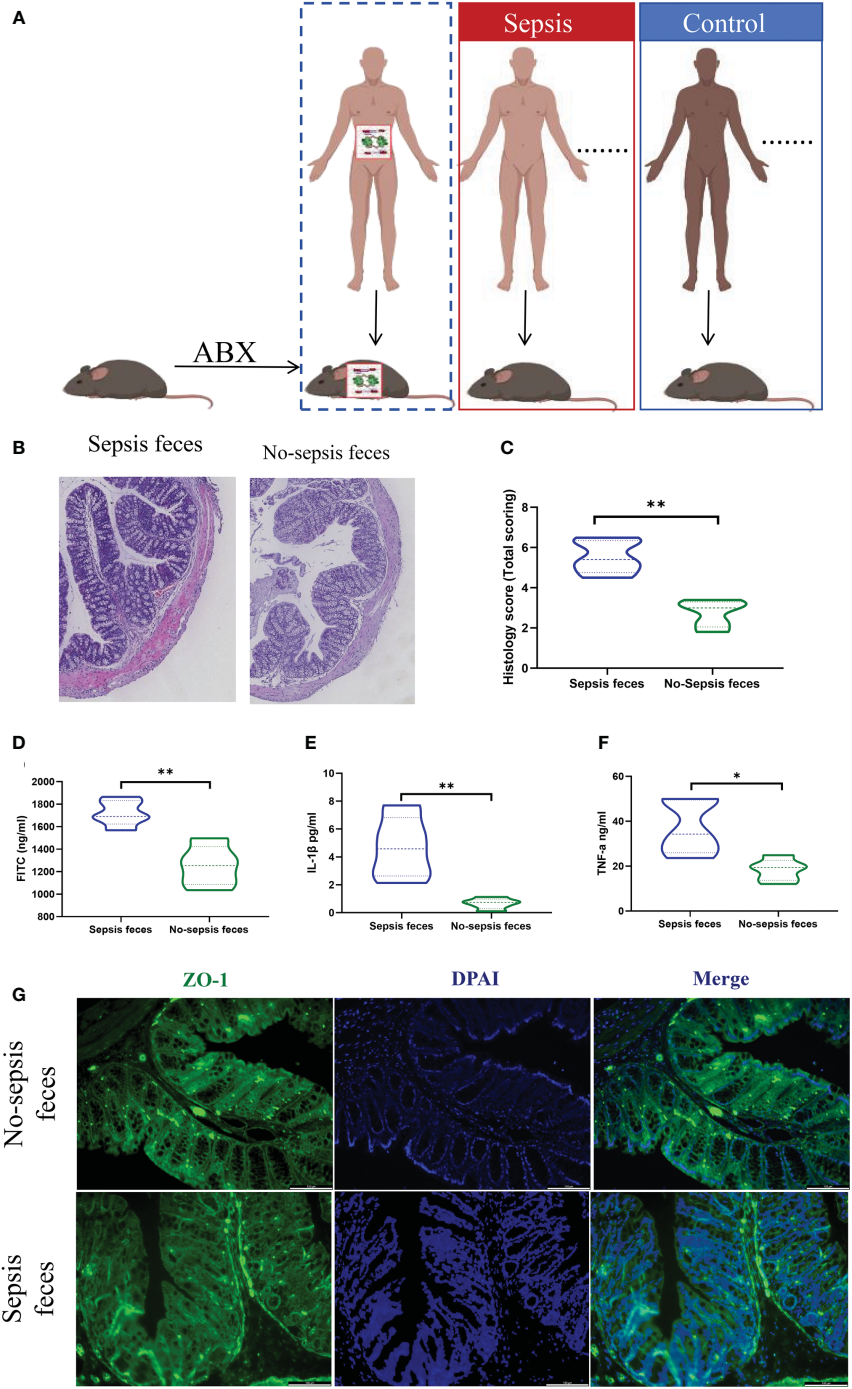
Gut microbiota colonization of sepsis lead to intestinal barrier damage. **(A)** Feces from the sepsis or the controls were transplanted into pseudosterile mice to establish a model of sepsis. Hematoxylin-eosin (HE) staining **(B)** and pathological damage scores **(C)** of intestinal tissue sections. Scale bar, 100 mm. **(D)** The fluorescein isothiocyanate (FITC)-dextran levels. **(E, F)** The express of plasma inflammatory factors (IL-1β and TNF-α) levels, **(G)** Immunofluorescence analysis of ZO-1 expression (ZO-1, green; nucleus, blue) in intestine. *P < 0.05, **P < 0.01..

The pseudosterile mice that received feces from the sepsis group and the control group were referred to as the sepsis feces group (n=13) and the control feces group (n=13), respectively. Subsequently, human feces from either the sepsis group or control group were resuspended in PBS at 0.125 g/mL and then transplanted into pseudosterile mice, once a day for a week, as illustrated in **Figure 2A**. All the mice had free access to food and water, and cecal ligation and puncture were performed after 1 week transplantation.

### 2.6 Histomorphological analysis

Tissue specimens from the intestines were preserved in 10% neutral-buffered formalin for 24 hours, encased in paraffin, sectioned into 4-micrometer slices, and then processed with hematoxylin and eosin (H&E) for histological staining. A pathologist blinded to group assignments assessed the intestinal damage using a light microscope (Olympus CX30, Japan).

### 2.7 Serum cytokine analyses

Mouse serum samples underwent centrifugation at 3,000 rpm and 4°C for 10 minutes, followed by storage at -80°C pending subsequent analysis. Levels of TNF-a, IL-1β, D-lactic acid levels were measured using ELISA kits (88–7064, Thermo Fisher, Austria; EK280/3–01, MuLTI SCIENCE, Shanghai; ab1174096, abcam, Shanghai) following manufacturer’s instructions.

### 2.8 Evaluation of FITC-dextran leakage

Intestinal permeability was evaluated through the detection of FITC-dextran leakage. After fasting for 8 hours, FITC-dextran (4 kDa) supplied by Sigma-Aldrich (USA) was dissolved in PBS. The mice were given an oral dose of 0.6 mg/g of body weight, with a concentration of 25 mg/mL. Blood samples of 400 µL were collected through retro-orbital puncture after 24 hours period. After centrifugation, the supernatants were combined with an equal volume of PBS. Fluorescence intensity readings for each diluted serum sample (100 µL) were taken using a multimode reader set to excite at 485 nm and emit at 528 nm, with a bandwidth of 20 nm. The amount of FITC-dextran was calculated by comparing it to a standard curve.

### 2.9 Immunofluorescent staining

The intestinal tissue sections were fixed in cold acetone post-freezing, and then permeabilized with a solution of 0.1% Triton X-100 and 0.1% sodium citrate at 4°C for 2 minutes. Subsequently, the sections were blocked with PBTB buffer comprising 0.2% Triton X-100, 0.2% bovine serum albumin, and 5% normal goat serum at room temperature for 30 minutes. This was followed by incubation with a mixture of primary antibodies anti-ZO-1 (1:100; ab228861, Abcam, UK) overnight at 4°C. After washing, the sections were then exposed to FITC-conjugated secondary antibodies at room temperature for 1 hour. Finally, the samples were counterstained with the nuclear stain DAPI and visualized using fluorescence microscopy.

### 2.10 Statistical analyses

Continuous variables were compared using the Mann-Whitney test or the Student t test as appropriate, while categorical variables were compared using the chi-square, normal approximation or Fisher’s exact test. Data were expressed as means with standard deviation (SD). SPSS software system version 20.0 (SPSS, Armonk, NY) and Graphpad Prism 6 (GraphPad Software, La Jolla, CA) software were used for statistical calculations. The correlations between the abundant OTUs and gut metabolites, disease severity were determined by Spearman correlation analysis. *P* < 0.05 was considered the threshold for significance.

## 3 Results

### 3.1 Intestinal flora diversity was significantly disordered in patients with sepsis

**Figure 3:**
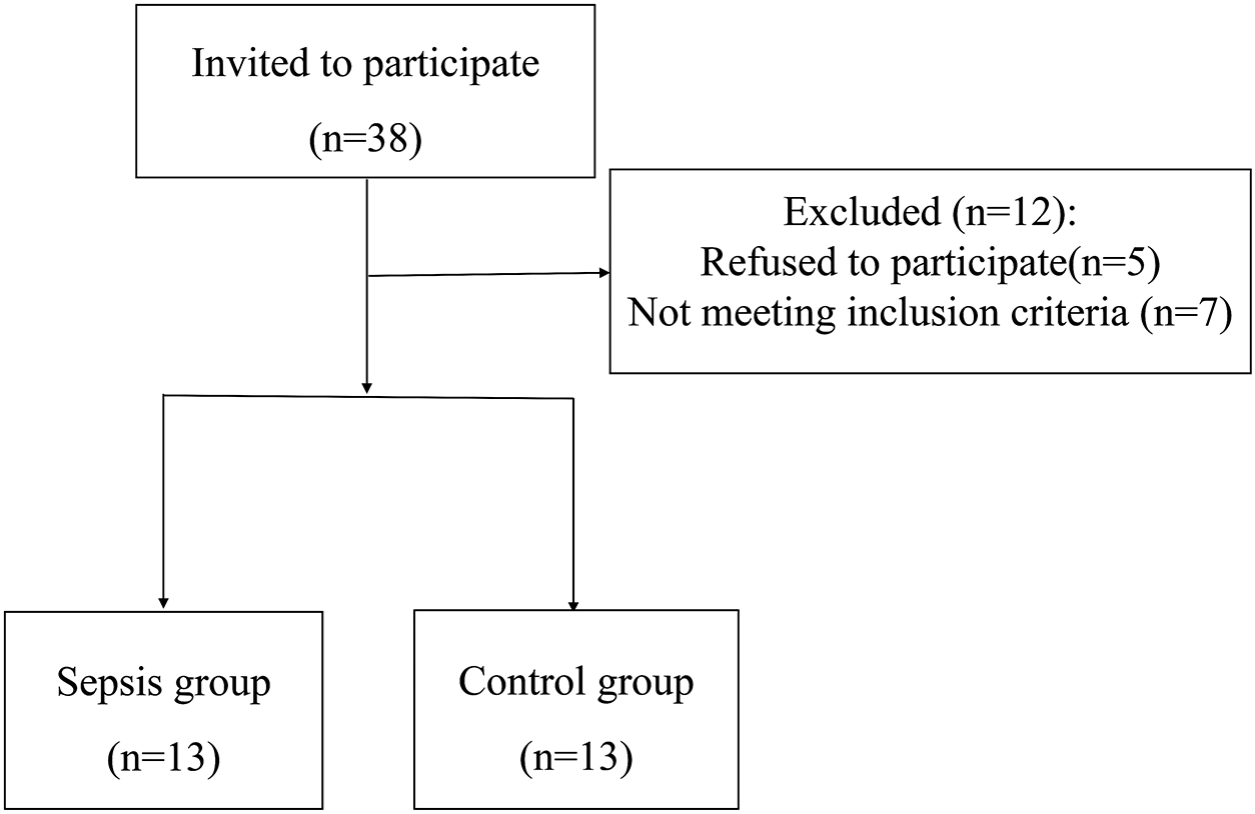
The flow diagram.

**Figure 4:**
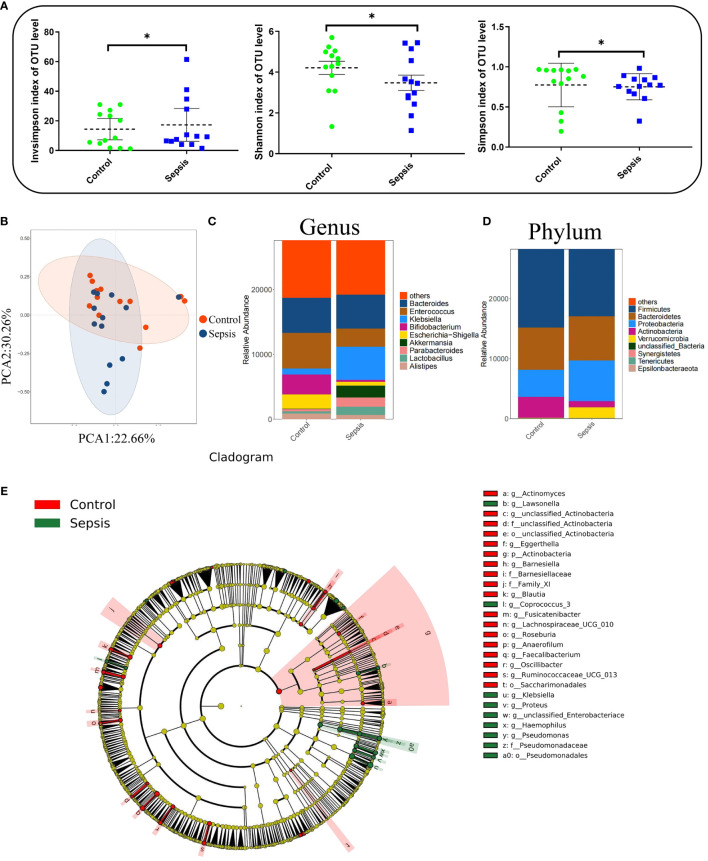
Intestinal flora diversity was significantly reduced in patients with sepsis. Human feces were collected from controls (n =13) and patients with sepsis (n =13) to investigate the bacterial compositions. **(A)** Alpha diversity analysis. **(B)** Principal coordinate analysis (PCoA) of 16S rRNA gene sequencing data. **(C)** Genus-level relative abundance. **(D)** Phylum-level relative abundance. **(E)** Cladogram were generated for items with a *P*-value < 0.05 (Kruskal-Wallis) and LDA score (log 10)≥ 2. *P < 0.05.

In total, 38 patients were initially screened for the study. Among them, 5 patients refused to participate, 7 did not meet the inclusion criteria. Finally, 26 patients were included in the study. There was no statistically significant differences were observed between the two groups in terms of baseline demographic and clinical characteristics.There were significant differences in white blood cell count, C-reactive protein (CRP), Procalcitonin (PCT), Sequential Organ Failure Assessment (SOFA)score, and Acute Physiology and Chronic Health Evaluation-II(APACHE-II) between the two groups (*P*< 0.01). **Table 1** and **Figure 3**. Studies have demonstrated a close relationship between intestinal flora and the pathogenesis of sepsis. Initially, we conducted a comparison of alpha diversity and beta diversity between the sepsis and control groups. Alpha diversity analysis revealed significantly lower InvSimpson, Shannon, and Simpson indices in the sepsis group compared to the control group, indicating reduced microbial diversity in sepsis, as depicted in **Figure 4A**. Furthermore, the sepsis group exhibited a distinct microbial structure compared to the control group, as shown in **Figure 4B**. Compared with controls, the relative abundance of *Bacteroides, Enterococcus*, and *Bifidobacterium* at the genus level was decreased in the sepsis group, while that of *Klebsiella* and *Akkermansia* was increased (**Figure 4C**). Furthermore, the relative abundance of *Bacillota, Bacteroidota*, and *Actinobacteria* at the phylum level was decreased in the sepsis group, while that of *Proteobacteria* was increased (**Figure 4D**) The primary taxa that differed between the groups were also identified. LEfSe analysis was used to explore taxonomic abundance, revealing significant differences between sepsis patients and controls. *Actinomyces* and *Eggerthella* were prevail in the control group; The sepsis group is dominated by *Lawsonella* and *eKlebsiella* (**Figure 4E**).

**Table 1:** Demographic characteristics of the cohorts.

Variables	Control group(n=13)	Sepsis group(n=13)	p valuea
Age(yr)	65.7±12.51	66.43±6.76	0.43
Sex, n (%)			
Male	6(46.15%)	8(61.53)	0.12
Female	7(53.85%)	5(38.47)	
IL-6	26.07±17.37	146.12±50.27	<0.01
APACHE-II score	3.97±1.61	16.19±4.91	<0.01
SOFA scoring	3.21±1.50	8.64±2.94	<0.01
CRP, mg/l	2.86±1.42	118.77±39.45	<0.01
PCT, ng/ml	0.05±0.02	4.94±1.16	<0.01
White blood cell count,10^9^/l	8.01±3.00	20.8±4.12	<0.01

CRP, C-reactive protein; PCT, Procalcitonin; SOFA, Sequential Organ Failure Assessment score; APACHE-II, Acute Physiology and Chronic Health Evaluation-II.

### 3.2 Gut microbiota colonization of sepsis lead to intestinal barrier damage

Comparatively, mice receiving fecal transplants from the control group exhibited reduced intestinal histopathological damage and lower scores than those receiving transplants from the sepsis group (**Figures 2B, C**). Additionally, the control group showed decreased intestinal barrier permeability (**Figure 2D**), lower plasma levels of inflammatory factors (IL-1β and TNF-α) (**Figures 2E, F**), and heightened protein expression of mechanical barrier markers like ZO-1 (**Figure 2G**) in contrast to the sepsis feces group.

### 3.3 Gut microbiota -derived metabolite L-valine was significantly reduced in the sepsis

**Figure 5:**
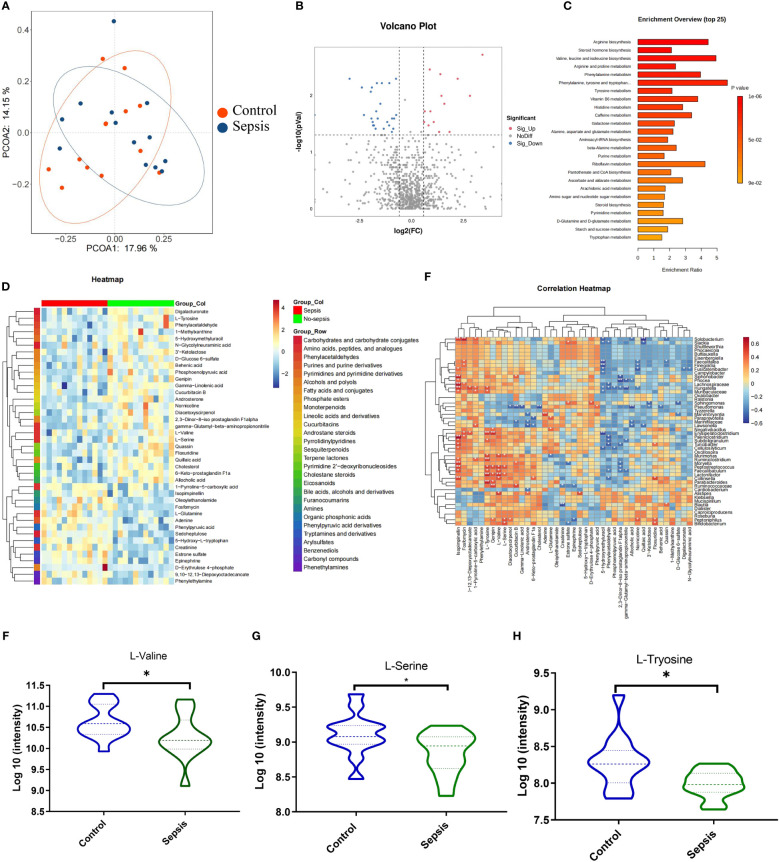
Gut microbiota -derived metabolite L-valine was significantly reduced in the sepsis. Human feces were collected from the controls group (n =13) and patients with sepsis (n =13) to investigate the intestinal metabolites **(A)** Principle coordinate analysis (PCoA). **(B)** Volcano plots analysis (Fold change >1.5). **(C)** KEGG pathway analyses of differentially abundant metabolites. **(D)** Heat map of differentially regulated metabolites. Spearman’s correlation analysis between the intestinal flora and fecal metabolites **(E)**. The relative expression of L-Valine **(F)**, L-Serine **(G)** and L-Tyrosine **(H)**. ^*^P < 0.05.

Metabolites generated in the gastrointestinal tract due to host-microbe interactions regulate the onset and progression of many diseases and autoimmune disorders (14). Besides, these metabolites have been used to diagnose or treat these conditions. Herein, PCOA plots scatter plots revealed that QC samples clustered together, consistent with high-quality metabolomics data (**Figure 5A**). According to *VIP*>1 and adjust *P* <0.05, with the employment of the volcano plot filtering method, several differentially expressed metabolites between the sepsis and control groups were observed (**Figure 5B**). Metabolite enrichment analysis revealed that the differentially abundant metabolites were significantly enriched in various pathways, including valine, leucine, and isoleucine degradation, tryptophan metabolism, tyrosine metabolism, purine metabolism, and steroid hormone biosynthesis pathways (**Figure 5C**). A heatmap of the top 50 metabolites revealed clear clustering of the differential metabolites in each group (**Figure 5D**). Relationships between the intestinal flora and fecal metabolites were evaluated by Spearman’s correlation analysis, L-Valine, L-Serine and L-Tyrosine were linked to beneficial bacteria, such as *Lactonifactor, Peptoniphilus* and *Parabacteroides* (**Figure 5E**). Among the valine, leucine, and isoleucine degradation pathway, we found that the relative expression of L-Valine, L-Serine and L-Tyrosine were lower in the sepsis goup (**Figures 5F–H**).

### 3.4 L-Valine was negatively correlated with the severity of sepsis

**Figure 6:**
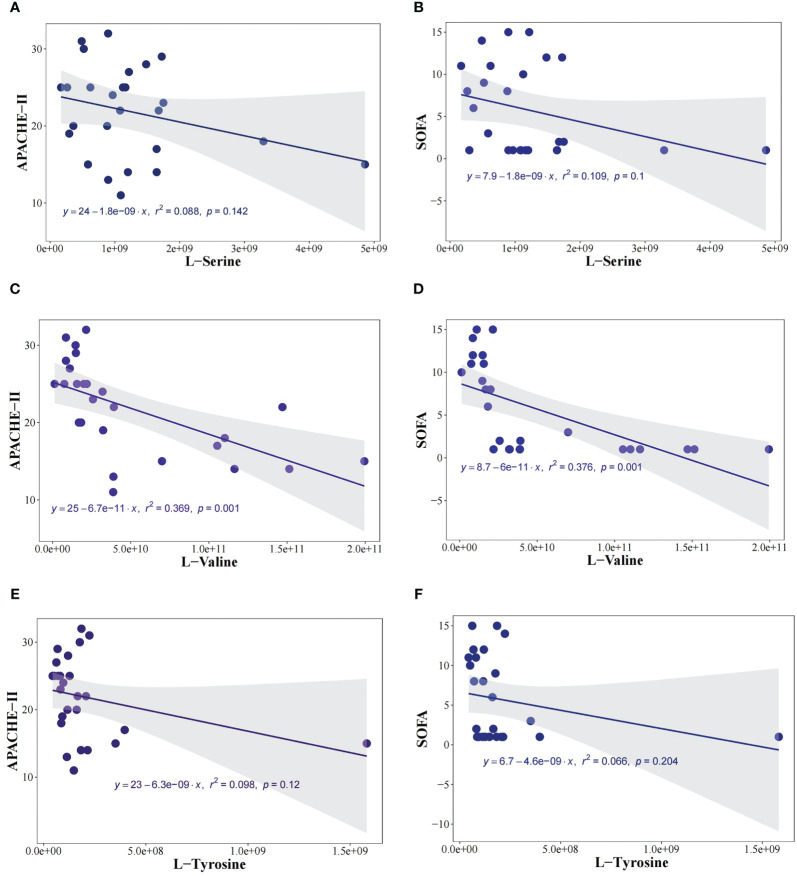
L-Valine was negatively correlated with the severity of sepsis. To investigate the relationship between metabolites (L-Valine, L-Tyrosine, and L-Serine) and the severity of sepsis, we analyzed the correlation between metabolites and Acute Physiology and Chronic Health Evaluation-II (APACHE-II) score, and SOFA score. The expression of L-Valine and APACHE-II (*r^2^* = 0.36, *P*<0.05), the expression of L-Tyrosine and APACHE-II (*r^2^* = 0.08, *P*=0.14), The expression of L-Serine and APACHE-II (*r^2^* = 0.09, P=0.12) **(A)**, the expression of L-Serine and SOFA score (*r^2^* = 0.11, P=0.10) **(B)**. The expression of L-Valine and APACHE-II (*r^2^* = 0.36, P<0.05) **(C)**, the expression of L-Valine and SOFA score (*r^2^* = 0.37, P<0.05) **(D)**. The expression of L-Tyrosine and APACHE-II (*r^2^* = 0.08, P=0.14) **(E)**, the expression of L-Tyrosine and SOFA score (*r^2^* = 0.07, P=0.20) **(F)**.

In exploring the relationship between metabolites (L-Valine, L-Tyrosine, and L-Serine) and sepsis severity, we conducted an analysis to correlate these metabolites with the APACHE-II score and SOFA score. The correlation results are as follows: Expression of L-Valine and APACHE-II score (*r^2^ = 0.36, P<0.05*); Expression of L-Tyrosine and APACHE-II score (*r^2^ = 0.08, P=0.14*); Expression of L-Serine and APACHE-II score (*r^2^ = 0.09, P=0.12*); Expression of L-Valine and SOFA score (*r^2^ = 0.37, P<0.05*); Expression of L-Serine and SOFA score (*r* ^2^ = 0.11, *P*=0.10); Expression of L-Tyrosine and SOFA score (*r^2^* = 0.07, *P*=0.20). These findings, as illustrated in **Figures 6A–F**, indicate a negative correlation between L-Valine and the severity of sepsis, suggesting that L-Valine levels may be inversely associated with sepsis severity.

### 3.5 L-valine protect against sepsis-induced intestinal damage in mice

A mouse model of sepsis was established to investigate the protective effect of L-valine against septic intestinal injury. In this study, sepsis caused 90% mortality rate in CLP mice. However, high-dose L-valine (10 mg/kg) treatment improved the 24 h survival rate of these animals compared with the CLP group(*P*<0.05)., while low-dose L-valine (5 mg/kg) did not improve the survival compared with the CLP group (**Figure 1B**). FITC, IL-1β, TNF-a and D-lactic acid levels were significantly elevated in the sepsis group. After 24 hours of treatment, L-valine reversed the levels (*P*<0.05) (**Figures 1C–F**). The degree of intestinal injury was assessed based on histological imaging. The imaging revealed that the mice in the control and L-valine groups had normal colonic mucosal structures, while mice in the sepsis group had ulceration and inflammatory cell infiltration. L-valine notably enhanced the colonic mucosal structure in the sepsis group, resulting in a significant improvement with only a few visible goblet cells observed (**Figures 1G, H**). Furthermore, there was an increase in the protein expression of mechanical barrier markers like ZO-1 (**Figure 1I**) as a result of L-valine treatment.

## 4 Discussion

In this study, we found that the diversity of intestinal flora is decreased in patients with sepsis compared to the control group. Gut microbiota from sepsis patients could trigger intestinal damage in antibiotic-treated mice through fecal microbiota transplantation experiments. L-valine levels was significantly decline in sepsis patients, and inverse correlation with the severity of sepsis. L-valine also was observed to alleviate sepsis-induced intestinal damage through mouse experiments.

Disruption of the gut microbiome increases the risk of sepsis and associated organic dysfunction (14). Due to the infectious nature of sepsis, antibiotic treatment can disrupt the intestinal flora. Therefore, in order to mitigate the impact of antibiotics and other medications on the flora, we collect samples from patients prior to their use, ensuring that the sequencing results of intestinal flora are not influenced by antibiotic or drug usage (15). Once sepsis occurs, several pathological conditions emerge including acute respiratory distress syndrome (ARDS) and delirium, further aggravating sepsis (16). In recent years, the emergence of fecal flora transplantation has offered a novel approach for the prevention and treatment of enteric-borne sepsis (17). In this study, sepsis contributed to intestinal dysbiosis, leading to a reduction in the diversity of this microbial ecosystem while increasing the abundance of harmful bacterial levels and decreasing the relative abundance of probiotic species. This dysbiosis may concur with alterations in metabolite production, potentially exacerbating intestinal inflammation. Further, in fecal microbiota transplantation experiments, it was observed that gut microbiota from sepsis patients could trigger intestinal damage in antibiotic-treated mice. The results strongly indicate that the colonization of gut microbiota in sepsis may indeed result in damage to the intestinal barrier.Intestinal microbes have the capacity to produce a diverse array of metabolites that can be taken up by host cells within the intestines, exerting either beneficial or detrimental effects on their physiological attributes (18, 19). Intestinal microbe-derived metabolites including short Chain Fatty Acids (SCFAs) and lipopolysaccharides (LPS) are important components of the intestinal mucosal immune barrier that still function in the context of respiratory tract infections (20). Metabolomics analyses demonstrated significant reductions in levels of beneficial metabolites in the sepsis group, such as L-valine. Further analysis showed that L-valine was negatively correlated with the severity of sepsis. Animal model experiments revealed that intestinal microbiota-derived L-valine alleviated inflammation and protected against sepsis-induced intestinal damage. There has been report in 2017 that valine can alleviate intestinal damage in sepsis in a mouse model (21), which consistent with the results of our study. To the best of our knowledge, there is a paucity of research investigating the potential protective effects of L-valine on the functionality of the intestinal barrier.Through Spearman correlation analysis, we found that L-valine is mainly positively correlated with the *Lactonifactor*. *Lactonifactor* is a Gram positive and anaerobic bacterial genus from the family of *Clostridiaceae* with one known specifications (*Lactonifactor longoviformis*). We also found a significant decrease in this bacterium in patients with sepsis; However, there is currently limited research on the function of *Lactonifactor*, and no previous studies have found its correlation with L-valine. Therefore, further animal experiments are needed to clarify the relationship between *Lactonifactor* and L-valine, as well as its protective role in sepsis induced intestinal injury. Therefore, exploring the impact of L-valine on mitigating sepsis-induced damage to the integrity of the intestinal tract holds promise as a novel therapeutic strategy.

In summary, these results demonstrate that gut microbiota can influence the development and progression of sepsis by altering the production of several bioactive and immunomodulatory metabolites. And the gut microbiome influenced host immune status through the release of L-valine to preserve intestinal barrier function. The potential limitations of this study should not be disregarded, such as the 16S rRNA gene is useful for identifying bacterial species on a broader taxonomic scale. However, for more exact identification, further genetic techniques, such as whole-genome sequencing may be required. This will be considered for further study.

## 5 Conclusion

In summary, the gut microbiota plays a crucial role in protecting against intestinal injury caused by sepsis. Moreover, there was a significant decrease in L-valine derived from the gut microbiota in sepsis patients, and negatively correlated with the severity of sepsis. Additionally, mouse experiments confirmed that L-valine could alleviate sepsis-induced intestinal damage. This study provided a new therapy for the treatment of intestinal injuries induced by sepsis.

## Data availability statement

The datasets presented in this study can be found in online repositories. The names of the repository/repositories and accession number(s) can be found below: http://www.hmdb.ca/, MTBLS7866 https://www.ncbi.nlm.nih.gov/, PRJNA905077.

## Ethics statement

The studies involving humans were approved by the Human Research Ethics Committee of the hospital, under the approval document numbered 2019-118. The studies were conducted in accordance with the local legislation and institutional requirements. The human samples used in this study were acquired from primarily isolated as part of your previous study for which ethical approval was obtained. Written informed consent for participation was not required from the participants or the participants’ legal guardians/next of kin in accordance with the national legislation and institutional requirements. The animal study was approved by The mice had free access to food and water. Animal experiments were approved by the Experimental Animal Ethical Review Committee of East China Normal University. The study was conducted in accordance with the local legislation and institutional requirements.

## Author contributions

ZW: Funding acquisition, Writing – original draft, Writing – review & editing, Conceptualization, Data curation, Formal Analysis, Investigation, Methodology, Project administration, Resources, Software, Supervision, Validation, Visualization. YC: Data curation, Formal Analysis, Writing – original draft. KS: Data curation, Methodology, Supervision, Validation, Writing – review & editing. YQ: Formal Analysis, Methodology, Writing – original draft. JT: Writing – original draft, Writing – review & editing, Data curation, Methodology, Supervision, Conceptualization, Formal analysis, Project administration, Validation. HZ: Writing – original draft, Conceptualization, Investigation, Software.
